# Artificial intelligence in cardiac magnetic resonance fingerprinting

**DOI:** 10.3389/fcvm.2022.1009131

**Published:** 2022-09-20

**Authors:** Carlos Velasco, Thomas J. Fletcher, René M. Botnar, Claudia Prieto

**Affiliations:** ^1^School of Biomedical Engineering and Imaging Sciences, King's College London, London, United Kingdom; ^2^Institute for Biological and Medical Engineering, Pontificia Universidad Católica de Chile, Santiago, Chile; ^3^Millennium Institute for Intelligent Healthcare Engineering, Santiago, Chile

**Keywords:** magnetic resonance fingerprinting (MRF), artificial intelligence (AI), cardiac MRF, multiparametric imaging, cardiac magnetic resonance (CMR)

## Abstract

Magnetic resonance fingerprinting (MRF) is a fast MRI-based technique that allows for multiparametric quantitative characterization of the tissues of interest in a single acquisition. In particular, it has gained attention in the field of cardiac imaging due to its ability to provide simultaneous and co-registered myocardial T_1_ and T_2_ mapping in a single breath-held cardiac MRF scan, in addition to other parameters. Initial results in small healthy subject groups and clinical studies have demonstrated the feasibility and potential of MRF imaging. Ongoing research is being conducted to improve the accuracy, efficiency, and robustness of cardiac MRF. However, these improvements usually increase the complexity of image reconstruction and dictionary generation and introduce the need for sequence optimization. Each of these steps increase the computational demand and processing time of MRF. The latest advances in artificial intelligence (AI), including progress in deep learning and the development of neural networks for MRI, now present an opportunity to efficiently address these issues. Artificial intelligence can be used to optimize candidate sequences and reduce the memory demand and computational time required for reconstruction and post-processing. Recently, proposed machine learning-based approaches have been shown to reduce dictionary generation and reconstruction times by several orders of magnitude. Such applications of AI should help to remove these bottlenecks and speed up cardiac MRF, improving its practical utility and allowing for its potential inclusion in clinical routine. This review aims to summarize the latest developments in artificial intelligence applied to cardiac MRF. Particularly, we focus on the application of machine learning at different steps of the MRF process, such as sequence optimization, dictionary generation and image reconstruction.

## Introduction

### Cardiac magnetic resonance fingerprinting

Cardiac Magnetic Resonance (CMR) imaging is widely accepted as a key non-invasive imaging technique for the evaluation of cardiovascular diseases (1). CMR enables comprehensive myocardial tissue characterization, evaluating specific parameters such as T_1_, T_2_ and T_1ρ_, relaxation times and extracellular volume (2–5). Hence, quantitative mapping of these parameters of interest has become a primary tool for diagnosis of cardiomyopathies. Conventionally, several MRI scans using different protocols are acquired sequentially to provide multiparametric quantification by encoding one parameter at a time. However, this methodology is time consuming and leads to long scan times, patient discomfort, and mis-registered parameter maps. In contrast, simultaneous quantification of multiple parameters would address several of these issues, making multiparametric quantitative CMR appealing for widespread use in the clinical routine.

**Figure 1:**
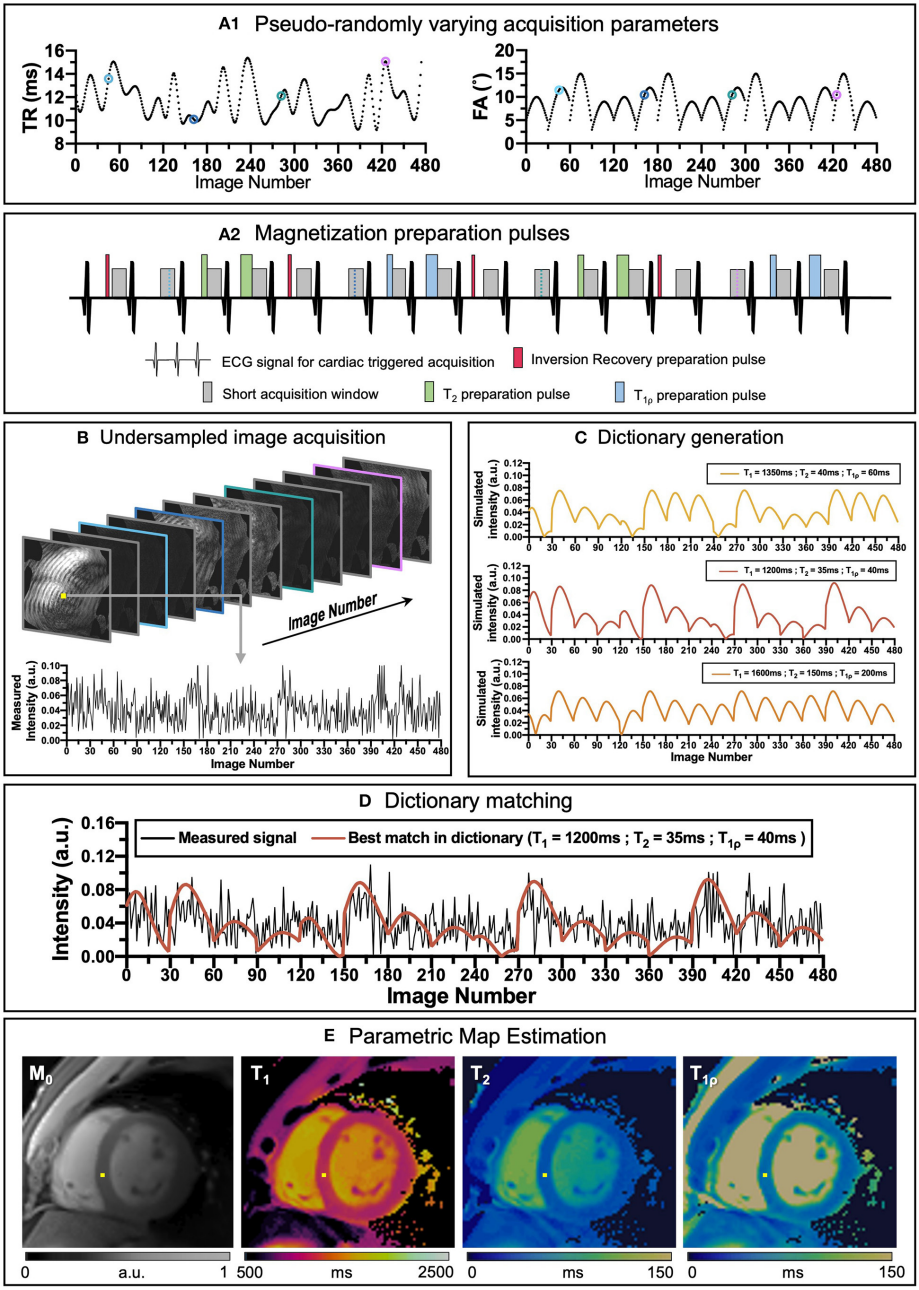
An overview of cardiac MRF framework. **(A1)** Repetition time (TR) and variable flip angles (FA) may be pseudo-randomly varied throughout acquisition. **(A2)** Magnetization preparation pulses are introduced to increment contrast weighing on the desired parameters (in this example Inversion Recovery, (IR pulses, in red), T_2_ preparation, (T_2_ prep pulses in green) and T_1ρ_ preparation, (T_1ρ_ prep pulses in blue) are included to encode T_1_ and T_2_ contrasts before some heartbeats). **(B)** Highly undersampled images are obtained, and **(C)** a subject-specific dictionary due to the unique cardiac rhythm during the scan is calculated in parallel. **(D)** Matching the temporal evolution of the signal measured with the dictionary will provide **(E)** inherently co-registered parametric maps of the scanned region. The different colored dots in **(A1)** correspond to different timepoints and contrasts **(B)** during the sequence.

Many simultaneous multiparametric approaches have recently been proposed to address these issues and provide co-registered multiparametric quantification, including Multitasking (6, 7), steady-state techniques with multiparametric encoding (8, 9), other free-running approaches (10, 11) and Magnetic Resonance Fingerprinting (MRF), (12). MRF has the potential to provide not only multiple co-registered parametric maps in a time-efficient manner but can also include additional model corrections [e.g., B_0_ (12), B_1_ (13), slice profile (14)]. Underlying MRF is the concept that each tissue has unique properties (such as T_1_ and T_2_) and thus unique signal evolutions for a given sequence. By varying several sequence parameters (such as flip angle, repetition time or magnetization preparation pulses) throughout the acquisition (see Figures 1A1,A2), unique signal evolutions (or “fingerprints”) for each combination of parameters of interest are created. Beforehand, a large dictionary (lookup table) of usually 10^4^ to 10^8^ parameter combinations and signal evolutions can be pre-calculated (Figure 1C), knowing the sequence details [using, for example Bloch simulations (15) or Extended Phase Graphs (EPG) (16)], and reutilized for the subsequent scans provided that the acquisition parameters remain unchanged. The measured signal evolution is then compared against the expected signal behavior *via* dictionary matching to simultaneously estimate the parametric maps in a voxel-wise basis, [respectively Figures 1D,E, (10)].

Although the original MRF work was proposed on brain MRI, the technique was rapidly implemented for other anatomic regions, including cardiac MRF (17). Compared to conventional MRF, cardiac MRF faces two main challenges derived for the inevitable heart motion that occurs during a scan. Firstly, since the heart is beating while the scan is being performed, the acquisition needs to be ECG-triggered, so that the acquisition window is always in the same cardiac phase. However, since the cardiac wave cannot be predicted, the dictionary must be specifically calculated for each subject once the acquisition has been performed to include the exact cardiac rhythm measured by the ECG. Secondly, cardiac and respiratory motions may affect MR image quality considerably. Therefore, scans are usually performed under breath-hold and on a short cardiac acquisition window (usually at mid-diastole), limiting the feasible scan time to the breath-hold duration and resulting in very highly undersampled data.

Despite these challenges, the field of cardiac MRF has experienced rapid growth. Many efforts have focused on improving its diagnostic potential by; extending the number of encoded parameters beyond just T_1_ and T_2_ [e.g., fat fraction (18), ${\text{T}}_{2}^{*}$ (19), T_1ρ_ (20)], optimizing sequence design (21), fast and robust dictionary generation (22–25) and advanced undersampled image reconstruction (26–28) among others. However, most of these techniques are computationally expensive using conventional methods, limiting their practical utility. There is still much room for improvement, and the integration of state-of-the-art developments in Artificial Intelligence (AI) and Deep Learning (DL) could help solve these and other challenges in cardiac MRF.

### Artificial intelligence and deep learning

**Figure 2:**
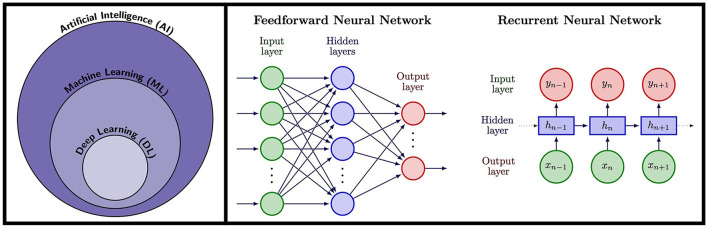
**Left:** Artificial Intelligence (AI) encompasses tasks performed by machines and computers that would normally require human intelligence. A subfield of AI is Machine Learning (ML), a technique whereby computer algorithms learn to perform a task from training data rather than requiring explicitly pre-programmed rules, allowing them to provide predictions for unseen examples. Deep Learning (DL) is a subfield of ML that uses artificial Neural Networks (NNs), modeled on neurons in the human brain, trained using data to provide predictions. **Right:** NN architectures used in cardiac MRF. Feedforward neural networks consist of an input later which could be for example a fingerprint in MRF, followed by a series of hidden layers, followed by a final output later that could output for example tissue parameters. RNNs are ideal for sequence data and take an input timepoint, along with an internal hidden state that encodes information from previous data in the sequence, thus incorporating memory of previous patterns in the sequence.

Artificial Intelligence refers to the ability of machines or computer algorithms to perform tasks that would typically require human intelligence (Figure 2). Machine learning (ML) is a subfield of AI where a model learns how to make predictions for a specific problem using relevant training data. In this way, the features of the model are learnt from relevant training examples without the need of explicitly pre-programmed rules, allowing the model to generalize and make predictions for unseen examples. The most advanced form of ML is DL, that uses multi-layered artificial Neural Networks (NNs) consisting of artificial neurons, inspired by biological neural networks. NNs are universal approximators (29), in theory able to approximate any Borel measurable function with a finite number of neurons. Thus, NNs can offer a more compact representation of complicated functions allowing for more efficient calculation. They are therefore ideal for cardiac MRF that involves complex acquisition strategies, scan-specific information, multi-dimensional image data dominated by noise, complicated reconstruction steps and computationally expensive calculations using conventional methods. Indeed, the ability of ML and DL has already been proven to be of great value in many domains of CMR imaging (30, 31), from image reconstruction (32–34) to diagnosis of cardiomyopathies (35, 36), reporting of cardiac function (37, 38), segmentation of cardiac CINE imaging (39, 40) and quantification of tissue parameters (41).

In traditional ML, measurable properties known as features are first extracted from relevant data. These features are then input into the model as training data, training the model parameters so that the model can find and generate accurate predictions of general underlying patterns using an optimization algorithm. The optimization algorithm measures the accuracy of these predictions using some quality measure, for example, the mean absolute error calculating the mean difference between predicted and ground truth values. The ability of the model to generalize is measured on a separate validation dataset. The accuracy of the predictions from the validation dataset are then used to optimize model parameters and prevent over-fitting to the training data. Finally, the model is evaluated on a separate held-out test dataset to simulate how the model will perform on unseen data. In medical imaging, models are often trained using supervised learning, where data is accompanied with ground truth labels. An example in cardiac MRF would be predicting signal evolutions labeled with ground truth parameter combinations and RR intervals (the time between successive R peaks determined from ECG data which itself is used to trigger the acquisitions in cardiac MRF). Models can also be trained in an unsupervised manner, where no labels are given. Autoencoders are one example, where a sparse representation of the data is learnt from unlabeled data and then this encoding is used to regenerate the input data, which can be useful for denoising applications. Self-supervised learning is a subset of unsupervised learning where supervisory signals are obtained from the data itself. For example, a cardiac motion algorithm can be trained without labels by using the motion estimation predictions to warp one cardiac phase to another and a loss term calculated between the now-similar images. Typically, there are a large number of features that the algorithm can use, and the accuracy of the model increases when more relevant features are used. The art of designing the optimal combination of features is called feature engineering but this can be a difficult task, usually requiring an experienced user and even so, the optimal features for a certain dataset are unlikely to be optimal for a slightly different one.

DL (42, 43) algorithms can learn features automatically from the dataset by themselves, removing the need to extract and select relevant features. NNs consist of multiple layers of connected nodes, called neurons, mimicking the behavior of the nervous system in humans. Each neuron is a mathematical function that takes one or more inputs and sums them with weights learnt during training, this is then passed through a non-linear activation function to produce an output. The NN is made up of layers of neurons with the outputs of one layer forming the inputs for the next layer and the depth of the network is given by the number of layers contained within the model. Recently, rapid advances in DL have been made due to the combination of the availability of large high-quality training datasets and tweaks to the architecture of NNs capable of extracting features. Alongside this, advances in GPUs for parallel computation and open-source libraries to construct and train DL algorithms have aided its adoption. There are a broad range of concepts and types of models within the field of DL, and a full description of them is outside the scope of this review. Thus, here only a small selection of terms of interest relevant to DL applications in CMR and more specifically in cardiac MRF will be briefly introduced.

#### Deep learning in cardiac MR

In DL for medical imaging and in particular for CMR imaging, input data is typically information obtained from the scanner, such as signal evolutions, k-space data or reconstructed images. The type of NN model these data are input into will affect the predicted outputs and the overall accuracy of the trained network. Hence, different types of network architecture are more suited to different types of data and problems. As an example, an image can be made up of patches of highly correlated data that form specific patterns or features such as edges, corners, ridges and blobs. A discrete convolution, a mathematical operation, can be used to filter these patches, hence a stack of convolutions can extract complex information and features from an image. Convolutional Neural Networks (CNNs) have been proven to be a powerful tool when working with imaging data and are widely applied in CMR. However, if the input is a set of temporal signal evolutions measured from a scan, a CNN may be less efficient than other types of networks better suited for long sequential datasets. Instead, recurrent neural networks (RNNs) such as long short-term memory (LSTM) networks can be used. These networks can track dependencies over a large number of time steps and remember previous inputs, making them more suitable for time-series predictions.

A good understanding of the type of input data and the nature of the problem is important to properly exploit AI for medical imaging. Accelerated (i.e., undersampled) CMR acquisitions lead to ill-posed inverse reconstruction problems. Many techniques, such as compressed sensing (44, 45) or low-rank-based reconstructions (26, 28), have been proposed to tackle these undersampled problems. However, the non-linear nature of the problems to be solved leads to long reconstruction times, in addition to the parameter tuning required for an optimal regularization. Several DL-based alternatives have been proposed to overcome these limitations and enable not only highly accelerated acquisitions in short reconstruction times (32, 33, 46–51), but also a wide range or AI-aided solutions for CMR segmentation (40) and analysis or outcome prediction (52, 53) among others.

Beyond quantitative CMR, many of the ideas in these studies have been successfully applied in MRF with the potential to be employed in cardiac MRF. Some of the most interesting ideas in AI along with their potential use in cardiac MRF are presented in the following sections.

## Artificial intelligence in cardiac MRF

**Figure 3:**
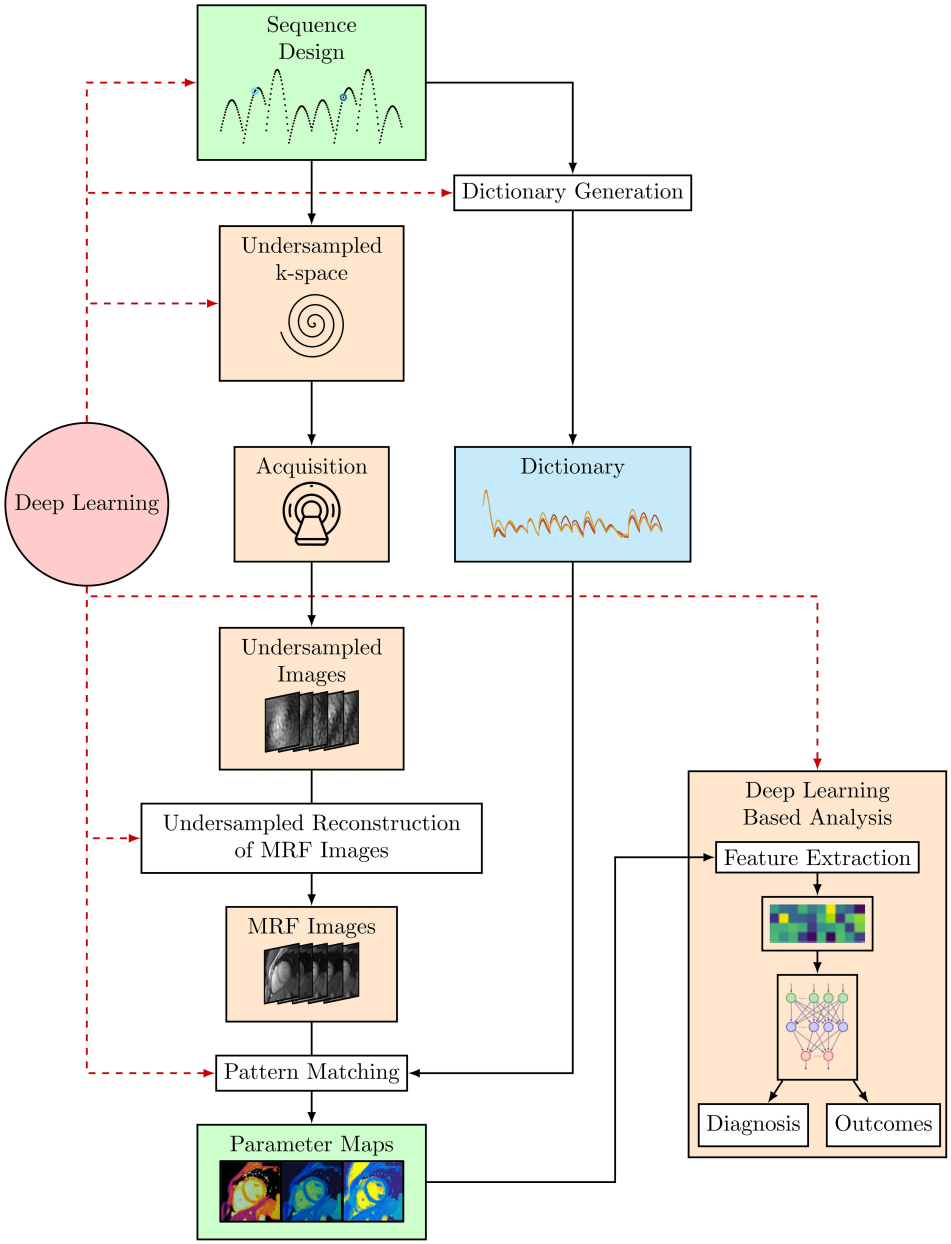
The cardiac MRF workflow from sequence design and optimization through to parameter map estimation and potential uses of cardiac MRF data, where DL-based analyses such as Radiomics can be used to provide a diagnosis or predict outcomes. Black arrows indicate the flow of steps taken in the cardiac MRF workflow. The red dashed arrows indicate steps where DL methods have or could be applied within this workflow.

Conventional cardiac MRF is a powerful technique for quantitative parameter estimation. However, the computational burden of dictionary generation and pattern matching grows exponentially with the number of parameters considered. Dictionary generation and pattern matching is especially challenging for cardiac MRF as it must incorporate information about subject-specific heart rate variability throughout the scan. In addition, the short acquisition times required for a cardiac MRF sequence to be feasible within a breath-hold means high acceleration factors must be used, leading to very highly undersampled k-space that must be reconstructed. Furthermore, since cardiac MRF sequences are frequently designed heuristically, it may be possible to further optimize sequence design to both shorten scan times and provide better discrimination between the parameters of interest. ML offers the ability to solve these problems and it has been or has the potential to be applied to each of these problems, to both speed up acquisition and reconstruction and optimize MRF sequences (see Figure 3).

### Dictionary generation

Dictionaries are conventionally generated using Bloch equation simulations or EPG calculations (16). The size of a dictionary and thus the time required to generate it scales with the sequence length (number of TRs) and the number of unique parameter combinations considered. Indeed, the size and thus time taken to simulate a dictionary grows exponentially with the number of tissue properties considered. For example, the dictionary for a 15 heartbeat T_1_, T_2_ cardiac MRF sequence contained 26,680 parameter combinations with 750 TRs and took 2.2 min to generate (54). However, the dictionary for a 16 heartbeat T_1_, T_2_, T_1ρ_ cardiac MRF scan contained signal evolutions for 253,000 parameter combinations with 480 time points (20) and can take ~15 min to generate using EPG simulations on a standard multicore CPU-based workstation.

As mentioned above, cardiac MRF differs from regular MRF as it uses ECG triggering to acquire signals during the same cardiac phase, across different heartbeats, to reduce artifacts from cardiac motion. This introduces a dependency of the measured signal evolutions on the subject-specific cardiac rhythm. Unlike MRF, where the pulse sequence is fixed and dictionaries can be generated ahead of time and used for all future scans, dictionaries for cardiac MRF must be calculated for each new scan using the measured RR intervals derived from ECG data recorded during the scan. This problem is further complicated when a more granular dictionary is required to reduce quantization errors, or as the number of modeled or encoded parameters is increased. This significant bottleneck presents a barrier to clinical adoption where long computation times for short scans hamper online parameter map generation in a fast-paced clinical workflow.

ML offers the promise of learning a surrogate model that can approximate the Bloch equations, turning dictionary generation into a straightforward pass through a NN. This provides the possibility of rapidly generating dictionaries in real-time, crucial for subject-specific cardiac MRF scans. Furthermore, rapid dictionary generation using ML makes it possible to quickly simulate dictionaries that consider larger numbers of parameters as well as aiding in applications such as sequence optimization where many different dictionaries must be generated. In the following sections we will explore in more detail studies where ML has been applied to the problem of dictionary generation in cardiac and non-cardiac MRF.

#### Fully connected feedforward neural network

A fully connected neural network has been proposed for generating dictionaries for a 16 heartbeat T_1_ and T_2_ cardiac MRF sequence (55). The network takes as input a 17-element vector consisting of T_1_ and T_2_ parameters plus 15 RR intervals. The input data, passes to two fully connected layers of 300 neurons, each followed by batch normalization and a ReLU activation function. The final output layer consists of 1,536 outputs corresponding to the real and imaginary components of the signal evolution of 768 TRs.

To model heart rate variability, the network was trained with dictionaries corresponding to 1,020 different cardiac rhythms. The mean of the RRs for each dictionary was varied from 40 to 120 beats per min (bpm) with a step size of 5 bpm. Noise was added to these RRs to simulate heart rate variability, with noise of standard deviation ranging from 0% to 50% of the mean RRs for a given dictionary. In total 4,392 T_1_ and T_2_ combinations were simulated for each dictionary resulting in ~4.5 million signal evolutions for training. In addition, missed ECG triggers were modeled with a 5% chance of each beat being a missed trigger, doubling the RR.

The network provided a significant time saving, generating a dictionary of 26,680 T_1_ and T_2_ combinations in just 0.8 s compared to 158 s using Bloch equations. Monte Carlo simulations were performed for a range of cardiac rhythms where for each rhythm, 500 dictionaries were generated using the network and the best-matching entry was found for a ground truth signal representative of healthy myocardium. The root mean square error (RMSE) for T_1_ and T_2_ for these simulations was found to be 2% or lower except for low heart rates with high variability. Further simulations, modeling up to 8 ECG missed trigger events showed the RMSE values to always be below 3%. The NN-generated dictionaries were also validated using phantoms and on *in vivo* cardiac mapping in healthy subjects. Maps constructed using the NN generated dictionaries appeared similar to those generated with dictionaries from Bloch equations, and there was 6.1 ms bias for T_1_ and 0.2 ms bias for T_2_.

Significantly, this network focused on cardiac MRF, considered a wide range of cardiac rhythms, mis-triggering events, and evaluated the generated dictionaries on phantom and *in vivo* data. However, the network only considered sequences encoding T_1_ and T_2_. Further improvements could extend this work to sequences encoding additional parameters. Furthermore, rather than training the network solely on simulated data, actual ECG data could be incorporated, thus exposing the network to cardiac rhythms and parameter combinations that may exist in real scans but may not be fully represented in simulated training data.

#### Generative adversarial networks

A different approach using generative adversarial networks (GANs) (56) has been applied to the problem of generating T_1_ and T_2_ dictionaries for MRF in the brain (57). This approach consists of a generative network that is given T_1_ and T_2_ tissue parameters, sequence parameters and pure random noise signals. It consists of 3 hidden layers of 128 neurons each followed by ReLU activation functions and an output layer with a hyperbolic tangent activation function with 1,000 output elements, mimicking a fingerprint. The discriminator network takes as input MR fingerprints, either generated by Bloch simulations or the generator network. It has a similar internal structure as the generative network, but it has an output layer with a sigmoid activation function, that represents a probability that the input fingerprint was simulated by Bloch equation simulations. The two networks are trained together, acting as two players in a min-max game, with the generator mimicking fingerprints to fool the discriminator and the discriminator improving such that it can distinguish between generated and ground truth fingerprints.

Fingerprints for a total of 5,970 T_1_ and T_2_ combinations were calculated using Bloch equations, each with 1,000 TRs. A 60:20:20 training, validation, test split was used to train and evaluate the model.

The GAN-MRF model introduced in Yang et al. (57) could generate a dictionary in 0.3 seconds with Python and Tensorflow, compared to several hours using Bloch simulations in MATLAB. Fingerprints synthesized by the GAN-MRF model were compared to those from Bloch equation simulations for white and gray matter and cerebrospinal fluid and provided a good match. A dictionary generated using the GAN-MRF model was used to reconstruct *in vivo* T_1_ and T_2_ maps and compared to benchmark maps reconstructed using a dictionary from Bloch equation simulations. The maps showed little difference, with RMSE of 0.55 and 2.66%, respectively for T_1_ and T_2_ maps, respectively. Additionally, the scalability of this method was tested by using coarser and finer dictionaries, compared to the grid of parameter combinations used in training, to reconstruct the *in vivo* maps. Again, good results were found with 1.69% and 6.39% RMSE, respectively for T_1_ and T_2_ maps for the coarse dictionary.

While the GAN-MRF model was only evaluated on brain MRF sequences, a similar model could be trained for cardiac MRF. However, GANs can be difficult to train due to the non-convex nature of the min-max problem and mode collapse, where the generator can learn a single pattern that seems the most plausible to the discriminator, thus fooling it. This is especially true when GANs are required to generate a wide variety of outputs, as is the case in MRF dictionary generation. Yang et al. (57) use regularization and a modified loss function to counter these affects. However, this requires a model-specific regularization parameter to be chosen. Also, convergence of the GAN during training will likely be more elusive for cardiac MRF due to increased variety in the fingerprints introduced by many additional degrees of freedom as a result of cardiac rhythm dependence.

#### Invertible neural networks

Invertible neural networks (INNs) (58) have also been employed to generate fingerprints from parameters in addition to predicting parameters from signal evolutions (59). Truly INNs such as NICE (60) and RealNVP (61) (the latter based upon real valued non-volume preserving transformations) are constructed of coupling layers, are invertible by design and have tractable Jacobian determinants and can thus easily be trained. Ardizzone et al. extended the RealNVP architecture to calculate posteriors for real-world inverse problems in natural sciences (58). Using this framework, an INN based upon a RealNVP (61) architecture, with two reversible blocks and two permutation layers, was trained on a T_1_ and fat fraction (T_1_-FF) MRF sequence (62) for diseased skeletal muscle with 175 TRs. Within each invertible block, two fully connected layers were used with 128 neurons, each followed by a ReLU and linear activation function. Dictionaries encoding Fat fraction, T_1, H2O_, T_1, fat_ and B_1_ were simulated with 396,000, 6,720 and 26,880 entries used, respectively for training, validation and testing. As well as providing good results for parameter matching when the INN is evaluated in the backwards direction (see Section Pattern matching), the estimated fingerprints were very accurate, with inner products between predicted and reference fingerprints > 0.997 for the majority of parameter combinations.

However, this INN was trained for a non-cardiac musculoskeletal sequence. Thus, it does not encode the additional degrees of freedom due to heart rate variability that is seen in cardiac sequences. Additionally, as with most inverse problems, while there is a direct mapping from physical parameters to signal evolutions, some information is lost in this forwards process. Thus, the backwards process (estimating tissue parameters from MRF signals) is often ambiguous, and a single fingerprint could correspond to a range of parameter combinations. Due to the cyclic nature of the INN and the fact no latent space was used to encode this information that is lost, large errors in this often-ambiguous backwards process can result in larger errors in the well-defined forwards process, hampering dictionary generation for some tissue combinations. Finally, dictionaries generated using this INN for non-cardiac MRF were not used to reconstruct *in vivo* data, nor was the parameter estimation part of the network evaluated on *in vivo* data.

#### Recurrent neural networks

RNNs, (see Figure 2) are capable of memorizing temporal structures within sequences and are therefore good candidates for dictionary generation in MRF. For instance, Liu et al. (63) propose a RNN as a surrogate model for dictionary generation for non-cardiac MRF. A novel feature of this network is that it is capable of modeling MRF signal evolutions resulting from different sequence parameters, in addition to encoding dependencies on tissue parameters. To achieve this, their RNN takes both tissue parameters and sequence parameters (such as repetition times, flip angles and sequence length) as inputs, and outputs MRF signal evolutions. Their RNN architecture consists of three stacked gated recurrent units (GRUs) and a linear layer, to generate the transversal magnetization and its derivates for every nth echo. This RNN can generate a dictionary three orders of magnitude faster than the snapMRF (25) GPU-accelerated EPG simulation package. As the RNN can rapidly generate both signal evolutions and derivative signals with respect to tissue parameters it is an ideal candidate for sequence optimization based upon Cramer-Rao lower bound (CRLB, see Section Sequence optimization). Liu et al. demonstrate that by using their RNN to optimize an MRF sequence, they improve the relative error in reconstructed *in vivo* brain T_2_ maps from 12.75 to 3.63%. The key advantage of this RNN is that it can generalize to predict fingerprints and their derivatives for different sequence parameters, compared to most networks that are trained for a specific sequence. However, the RNN was ~24 times slower generating dictionaries than the network proposed by Hamilton et al. (55). Extending the RNN to encode cardiac rhythm dependence for cardiac MRF would likely require significantly more training data, longer training times and a model with more layers and parameters.

### Undersampled reconstruction of time-series MRF images

The next significant step in the MRF framework is the reconstruction of acquired undersampled data. In the case of cardiac MRF, there is a need to execute the sequence in a short time (so it can be run within a breath-hold) and within short acquisition windows (so there is minimal corruption by cardiac motion). This usually translates into elevated acceleration factors, which leads to very highly undersampled k-space data of the order of 10x-10^2^x and produces severely aliased images at each timepoint. This can be alleviated by exploiting redundancy in the acquired data, as in Cruz et al. (64, 65), where a regularized low-rank high-dimensional patch-based tensor is used to improve the reconstructed image quality noticeably despite high undersampling factors, although this adds computational expense.

Cardiac MRF could benefit from recent advances in Deep MR quantitative imaging where end-to-end DL-based approaches (66–71), reconstructing images and maps from k-space data, have been explored. Although the literature on this topic is vast, there are reviews that summarize much of this work (41, 72). Some of the most relevant studies are mentioned here.

For instance, Jeelani et al. (73) propose a CNN, where spatial and temporal information is exploited for fast end-to-end myocardial T1 mapping, using MOLLI weighted images as the network's input. Cheng et al. (74) propose an unsupervised end-to-end network for T_1?_ mapping of knee cartilage. The network has a compressed sensing loss function and an unrolled approach with two chained networks, one to generate reconstructed contrast-weighted images and another for map generation. Their approach generates both maps and contrast-weighted images from undersampled k-space data and incorporates data consistency, the sparse prior of the image and prior information provided by the signal model. By using a compressed sensing loss function and training in an unsupervised manner, their network eliminates the need for fully sampled training data needed for supervised approaches, as is the case for MANTIS (67). A similar approach could be used for cardiac MRF by replacing the map generation network with one that incorporates cardiac rhythm dependencies.

The reconstruction process within cardiac MRF could also benefit from the advances in dynamic MR reconstruction. For instance, Qin et al. (75) propose a 3D Convolutional recurrent neural network (CRNN) able to faithfully produce CINE image reconstructions from 9x undersampled data, by learning from information propagated along time dimension and also through iterations.

A large number of scans are usually required for optimal training of a NN, especially in multiparametric CMR reconstruction with its dependency on subject-specific cardiac rhythms during acquisition. Ulyanov et al. (76) propose Deep Image Priors, a possible solution to this drawback. In their work, they show that the structure of deep convolutional generator networks can sufficiently capture enough image statistics prior to any learning. Hence, inverse problems such as MR reconstruction can be solved by randomly initializing a NN's parameters and searching for the optimal parameters to accurately reconstruct an image from a single degraded input image on an image-by-image basis. As a result, this eliminates the requirement for a large number of scans for training data. This method has already been successfully applied to dynamic MR reconstruction (77) and T_2_ mapping from undersampled data (78). Recently, low-rank subspace modeling has been combined with a deep image prior for a cardiac MRF sequence in a self-supervised framework (79). This results in improved quality of reconstructed maps, reduced noise and aliasing artifacts, enabling the sequence to be modified to both improve scan efficiency and reduce motion artifacts.

### Pattern matching

Conventionally, pattern matching or template matching for MRF involves an exhaustive search of the dictionary receiving as input the reconstructed time-series MRF images. For a given voxel, the dot product between the measured signal and each of the dictionary entries is calculated. In this way, a measured fingerprint is matched to the most similar signal evolution in the dictionary, and the voxel is assigned the parameter combination corresponding to that entry. While this method can find the globally optimal match from all simulated fingerprints, in the case of cardiac MRF it first requires a subject-specific dictionary to be generated which can require large amounts of storage. Also, both the dictionary generation and dot product matching become prohibitively computationally expensive as the size of the dictionary grows with the number of parameters encoded in the sequence or as the sampling along each dictionary dimension becomes more granular. Dot product matching can also result in quantization errors as parameter estimation is limited by the discrete step sizes used in simulating the dictionary.

Improvements to conventional pattern matching have been proposed. One approach, tested for dictionary sizes of 10^4^ to 10^5^, snapMRF (25), parallelizes dictionary generation and pattern matching on the GPU and results in 10–100 times speed-up for pattern matching compared to other open-source packages. Singular value decomposition (SVD), where the dictionary and observed signals are compressed in the time domain and matching is then performed in the compressed space, without sacrificing signal-to-noise ratio (SNR), has been proposed. Accelerated dictionary search methods, including fast group matching (80) and the Fast Library for Approximate Nearest Neighbors (FLANN) (81), first group similar dictionary entries and compare measured signals to representative signals for these groups, which are successively pruned. In this way only a portion of the total dictionary entries are searched over, and fast dictionary matching proves to be almost two orders of magnitude faster than an exhaustive search of the dictionary. Similarly MRF-ZOOM (23), iteratively refines its parameter estimation by searching over a more coarse version of the dictionary.

However, in the case of cardiac MRF, these methods still require a subject-specific dictionary to be generated, which itself is a significant computational bottleneck. Further to this, SVD requires the dictionary to be compressed and accelerated dictionary matching techniques require some grouping of dictionary entries, both of which must be repeated with each new scan and thus dictionary for cardiac MRF. Finally, all these methods depend on a dictionary and as a result they are limited by the discrete sampling used when generating the dictionary.

DL-based methods additionally offer the promise of completely bypassing the dictionary generation step, and to generate MRF parametric maps in real time with continuous variables. A NN can be trained as a surrogate model to learn the mapping from measured signals to tissue properties. These methods can transform the pattern matching step from an optimization-based problem, the complexity of which grows exponentially with the number of parameters modeled, to a much faster forward pass through a network. DL methods either work on a fingerprint-wise basis, reconstructing individual fingerprints, or on a spatially regularized basis, reconstructing a small patch of data and leveraging information from neighboring fingerprints that is likely correlated. They are typically trained on noiseless data from dictionaries or acquired *in vivo* data where it is also possible for NNs to reduce the amount of noise and aliasing in reconstructed maps compared to conventional dictionary matching. In the following sections we will discuss some of these different approaches for cardiac MRF and non-cardiac MRF.

#### Fully connected networks

Fully connected neural networks (FCNN) have been proposed to perform pattern matching on a voxel-wise basis for cardiac MRF (82), as well as brain, liver and prostate MRF (83, 84) for T_1_ and T_2_ mapping. The network architectures for all these approaches have an input layer consisting of either the magnitude fingerprints or concatenating the real and imaginary components of the fingerprint. These are followed by a series of hidden layers, each with activation layers and a final output layer with sigmoid activations for the regression outputs, corresponding to the parameter values. The DRONE (83) network for brain MRF and the network for brain, liver and prostate (84) (hereafter referred to FCNN2) have just 2 and 3 hidden layers, respectively (300 and 256 neurons each layer, respectively). However, the FCNN for cardiac MRF (82) must also take as input 10 RR intervals, to model the dependency of the measured sequence on the subject's cardiac rhythm during the acquisition. As a result, the network is much deeper consisting of 18 hidden layers (300 neurons each) with skip connections every 4 layers, beginning after the first hidden layer, to prevent vanishing gradients during training.

The DRONE network was trained with 69,000 EPG-generated signals with Gaussian noise added to the simulated signals to promote robust learning. It was evaluated on *in vivo* data reconstructed using a sliding-window approach (85), which removes most of the artifacts due to the spiral undersampling used. Other studies focus on directly training on signals that include artifacts due to the non-Cartesian undersampling artifacts from spiral trajectories used in cardiac MRF. In an attempt to make more realistic training data in a scalable manner, without acquiring *in vivo* data, the cardiac MRF FCNN (82) used pseudo-noise from a pre-computed library generated before training. The pseudo-noise library was generated by simulating the data acquisition of random maps using a spiral k-space trajectory and subtracting fully sampled reference images from the undersampled images. The noise patterns were then randomly scaled, phase shifted and added to the simulated signals with no noise which were also randomly phase shifted, resulting in simulated signals with pseudo-noise. In total, 8 million signal evolutions were simulated across 4,000 different cardiac rhythms, and these were combined with 1.8 million pseudo-noise samples. A similar approach for generating pseudo-noise for artifact patterns was used to generate training data for the FCNN2 (84), making it possible for both of these networks to take MRF images with undersampling artifacts as input.

**Figure 4:**
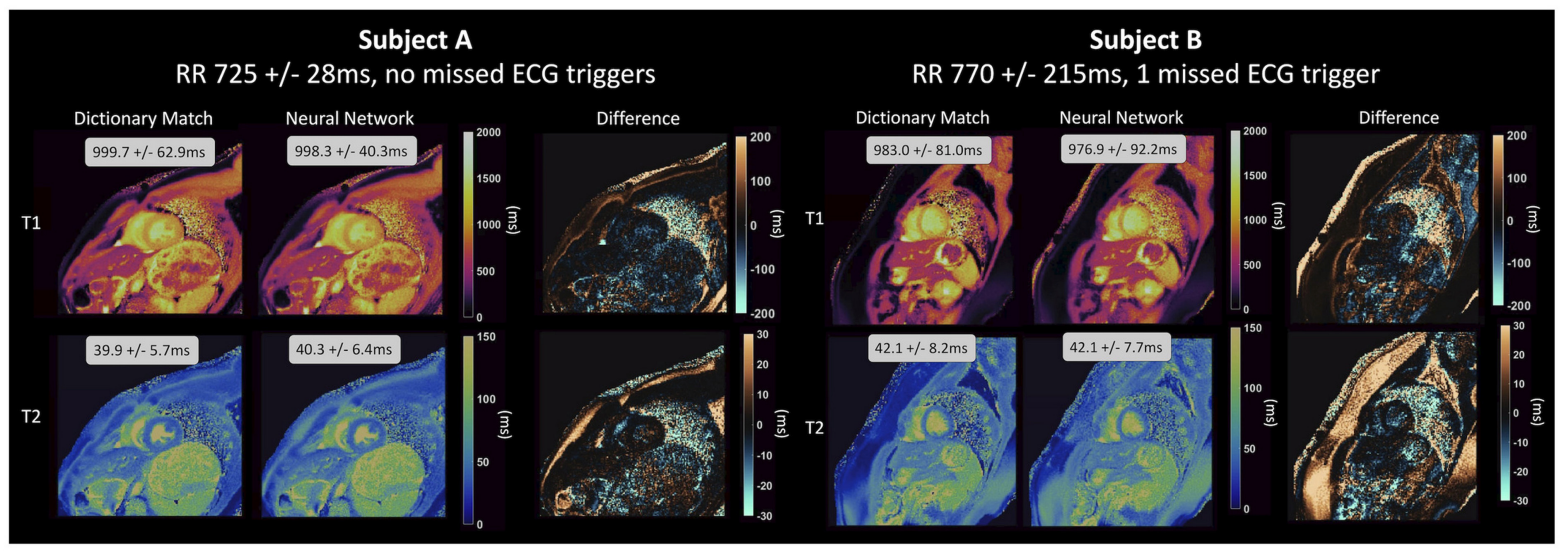
Parameter maps generated by a NN for cardiac MRF from Hamilton et al. (82) for two healthy subjects and compared to maps generated using dictionary matching. The feedforward network with skip connections that was used takes the real and imaginary components of the fingerprint and the RR intervals as input and outputs parameter estimates for T_1_ and T_2_ on a fingerprint-wise basis. The network produces accurate parameter estimations for different cardiac rhythms, even for subject B, with a variable heart rate and 1 missed ECG-trigger.

The DRONE network achieved relative errors of <3% when evaluated on simulated data with no noise but this increased significantly in Monte Carlo experiments where the SNR was varied, climbing to ~15% and ~48%, respectively for T_1_ and T_2_ at the lowest SNR. For the models trained on undersampled data, FCNN2 performed much better when trained using pseudo-noise and achieved *R*^2^≥0.98 in phantom experiments and good results on *in vivo* data >200 times faster than conventional dictionary matching. Equally, as is shown in Figure 4, the cardiac MRF FCNN achieved good results, with *R*^2^ = 0.93 for T_1_ and *R*^2^ = 0.95 for T_2_ and could quantify gridded sections of images in <400 ms compared to 10 s with conventional methods and without the need of a subject-specific dictionary that typically takes 4 min to generate.

#### Convolutional neural networks

As mentioned before, CNNs are commonly used in image and pattern recognition and work by performing convolutions using filters learnt during training. Therefore, they are excellent candidates for MRF pattern recognition, where fingerprints have repeating patterns and shapes encoding tissue parameters, and fingerprints are spatially correlated due to tissue structure and undersampling patterns in k-space.

One-dimensional CNNs have been proposed for brain MRF (86, 87). A 1D residual CNN (87) was proposed consisting of two 1D convolutional layers followed by 4 residual blocks, with 1D CNN architecture and short-cuts, followed by a max-pooling layer and two fully connected layers to give the parameter outputs. The advantage of this network is that the CNN architecture can learn patterns in the input signals, while residual blocks allow the model to avoid vanishing gradient problems as the model becomes sufficiently deep to effectively learn the mapping from measured signals to parameters. The network is trained using dictionary generated sequences and a low-rank prior is exploited for signature restoration of *in vivo* data before it is input into the network. The network outperforms dictionary matching on synthetic maps without undersampling as well as maps with 15% undersampling, where it provides comparable results to a conventional low-rank method (88). Importantly, the network produces T_1_ and T_2_ maps in 1.6 seconds, 56 times faster than dot product dictionary matching.

A further refinement of this method, HYbrid Deep magnetic ResonAnce fingerprinting (HYDRA) (86) inspired by self-attention and non-local NNs, modifies the architecture of the previous network to include non-local operations. The non-local operations capture long-range dependencies of the signal in the temporal dimension, thus extracting global features which would not be captured by convolutions alone that process one local neighborhood at a time. Importantly, parameter estimation for HYDRA is continuous and errors for predicted T_1_ and T_2_ are as small as ~0.2 ms, compared to errors of up to 4.5 ms due to discrete sampling every 10 ms in the case of dictionary matching. Song et al. demonstrate for noise-free synthetic data that HYDRA outperforms dictionary matching and continuous methods including the DRONE FCNN (83) and a standard 1D CNN, with HYDRA having the smallest deviations and bias. When applied to *in vivo* data where signature restoration using a low-rank prior is performed, HYDRA outperforms dictionary matching and other DL based methods for fully sampled, 15% undersampled and especially for 9% undersampled data with variable density spiral trajectories. For undersampled data, HYDRA is also 4.8 times faster than a competing low-rank method for parameter map generation (88).

Spatially regularized or spatiotemporal CNNs have also been proposed for MRF (89, 90). These networks are motivated by noisy reconstructions arising from fingerprint-wise reconstructions, the fact that neighboring tissue properties are likely correlated and that undersampling in k-space leads to the signal from one pixel being distributed to several other pixels. To determine parameter values at each voxel, these networks take MRF image patches as input, a square grid of fingerprints centered on the voxel. Initially, a spatiotemporal CNN using 5x5xT image patches (5x5 image patches in image dimension and length T corresponding to the size of the fingerprints in the temporal dimension) was explored for T_1_ and T_2_ brain MRF (89). The approach in training and evaluation of this network differed from most studies, in this case ground truth maps derived from separate T_1_ and T_2_ scans were used instead of deriving these maps from dictionary matching using the MRF scans. A sliding-window reconstruction (85) was used to partially reconstruct the data and to obtain MRF images for input into the network. Applying this method demonstrated that the spatiotemporal CNN outperformed the DRONE (FCNN) (83), a 1D CNN and spatiotemporal dictionary matching (91), achieving the lowest RMSE and producing less noisy maps than the fingerprint-wise networks.

**Figure 5:**
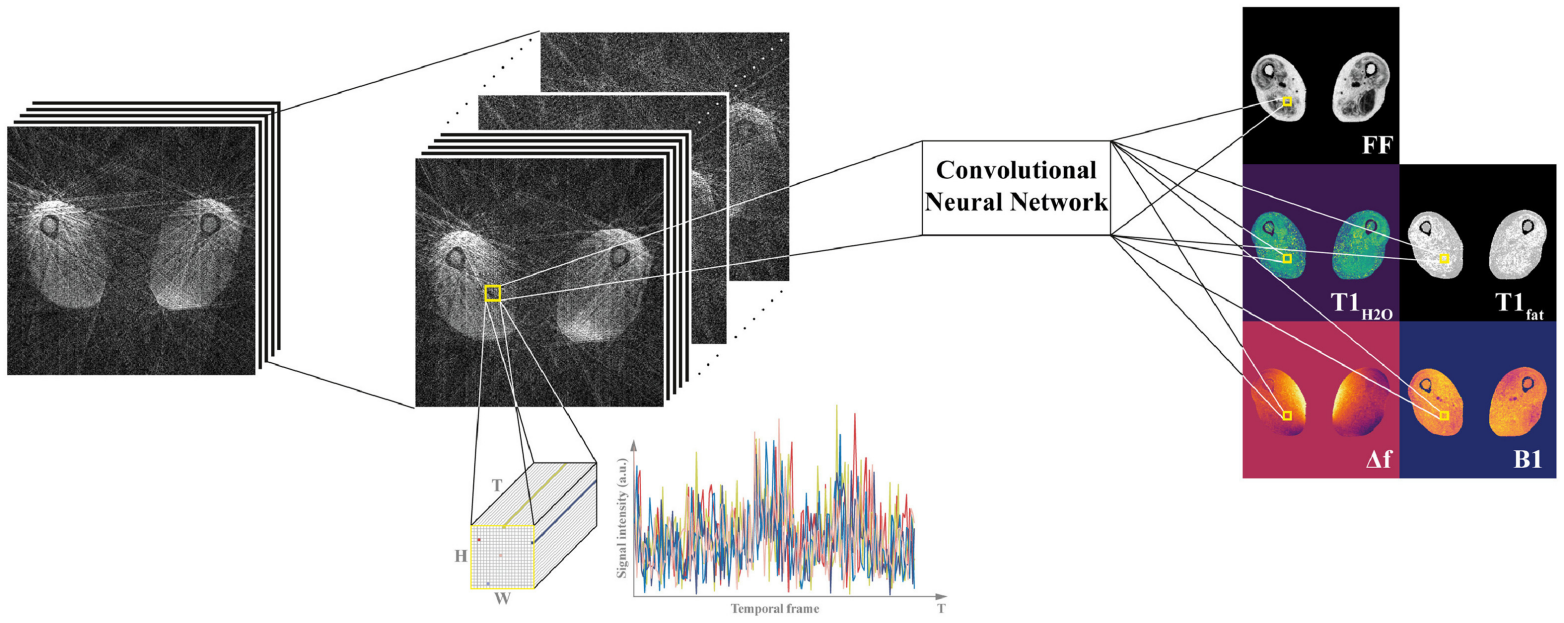
Spatially-regularized convolutional neural network from Balsiger et al. (90). In addition to the temporal information from each fingerprint, for each voxel a HxWxT patch of undersampled image data is used to calculate the parameter maps [in their work, Balsiger et al. (90) employ a patch size of H = W = 15]. The network achieves improved accuracy by incorporating spatial regularization in the map generation process from undersampled image data.

This spatially-regularized network was extended by Balsiger et al. (90), this time with a HxWxT image patch (see Figure 5) using a database of 164 MRF scans for a T_1_-FF sequence (62) that gives 5 parametric maps. A non-uniform fast Fourier transform (NUFFT) (92) was used to transform the data to image space which led to some undersampling artifacts in the input data to the network. This CNN achieved the best reconstruction compared to dictionary matching, a fingerprint-wise RNN (93) and a spatially regularized network (94) without introducing artifacts. Additionally, this method generalized to anatomical regions not previously seen during training.

A network combining residual channel attention blocks (RCABs) and a U-Net (RCA-U-Net) has been also investigated for brain MRF (95). The network improves upon U-Net architectures by including RCABs at each layer of the U-Net. These include channel attention blocks to focus on the most informative features for parameter quantification, and residual skip connections to allow more efficient flows of information within the network. The U-Net itself includes 3 down-sampling and 3 up-sampling layers to extract spatial information at different scales. The network is trained on undersampled *in vivo* brain scans. Training data input into the network are first head-masked and then passed through a compression network for feature extraction and dimensionality reduction. The performance of the RCA-U-Net was compared to conventional dictionary matching, SVD matching (22), a 1D-CNN (96) and spatially-constrained quantification network (94). For the high acceleration rates used (8x and 16x), the RCA-U-Net achieves improved accuracy compared to all the other state-of-the-art methods with relative errors of <2%. In particular, the RCA-U-Net provides accurate T_2_ estimation (1.4% error) for standard scans with an acceleration factor of 16 when compared to conventional dictionary matching (6.2% relative error).

#### Recurrent neural networks

RNNs, with their ability to memorize temporal structures, have also been employed for pattern matching for brain T_1_ and T_2_ MRF sequences (93, 97) where signal evolutions evolve continuously, there is redundant information and patterns are repeated. Both Oksuz et al. and Hoppe et al. use Long Short-Term Memory (LSTM) RNNs followed by fully connected layers which lead to outputs for the parameter values on a voxel-wise basis. Oksuz et al. also use GRUs equivalent to RNNs. The RNNs provided good results, with the LSTM from Hoppe et al. (97) outperforming a comparable CNN (96) method on *in vivo* data and the networks in Oksuz et al. providing lower mean absolute errors than the DRONE FCNN (83), a 1D CNN and conventional inner product matching on EPG generated signals.

#### Invertible neural networks

INNs have been proposed for a wide range of inverse problems in natural science (58). Inverse problems, such as cardiac MRF parametric mapping generation, typically involve determining physical parameters (*x*) from a set of measurements (*y*). While the forward process from parameters to measurements (Bloch equation simulations) is well understood, the backward process, or inverse problem, from measurements to parameters (parametric mapping generation) is often ambiguous. Ideally, cardiac MRF sequence design would make the inverse problem as unambiguous as possible, but this becomes more challenging due to artifact noise from undersampling and as the number of encoded parameters increases. INNs are unique in that they jointly optimize the well-defined forward problem and the ambiguous inverse problem using the same network and weights. During training, a latent space (*z*) is introduced that encodes information lost in the forwards process, which then aids the network in the inverse process, where the latent space is sampled over ([[*y, z*] → *x*]), allowing it to disentangle ambiguous cases. It has been demonstrated by Ardizzone et al. (58) that for INNs, learning the forwards process and latent space dramatically improves the accuracy of parameter estimation, compared to learning the backward process alone.

Balsiger et al. (59) implemented an INN to perform both dictionary generation and pattern matching on a voxel-wise basis for musculoskeletal MRF sequences. In their implementation training data simulated using the Bloch equations was augmented with Gaussian noise and no latent space was used in the network. The INN consisted of two reversible blocks, each followed by permutation layers, with each reversible block using fully connected layers with 128 neurons each. Their INN accurately generated fingerprints (inner product > 0.995) and significantly outperformed non-invertible networks (83, 86, 93, 97) for the pattern matching step, achieving mean relative errors for parameter estimation ranging from 2 to 8%. Inferring parameter values for 1,000 fingerprints took just 50 ms with the INN, comparable to other ML based methods.

INNs offer multiple avenues for improvement which could aid in using them for parametric mapping generation for cardiac MRF. Conditional INNs could be used for cardiac MRF where both the forward and backward processes in the INN could be conditioned on subject-specific RRs. Additionally, the invertible blocks within INNs can consist of any network architecture and can therefore be adapted to use convolutional networks to operate on image patches rather than on individual voxels. Finally, by sampling the latent space, INNs can provide marginal posterior distributions *p*(*x*|*y, z*) for each parameter of interest. This could give insight into the uncertainty of parameter estimation for cardiac MRF, the correlation between marginal posterior distributions across parameters and innately measure whether the signal from certain regions is multi-modal, due to combinations of different tissues.

#### Complex-valued neural networks

Typically, the real and imaginary components of complex-valued MRF signals are concatenated or input as 2-channels into real-valued NNs. However, this approach neglects the phase information and may lead to poorer reconstructions had the phase been considered. To rectify this problem, Virtue et al. (98) proposed a complex-valued NN for parameter estimation in MRF that includes a complex cardioid activation function sensitive to the input phase rather than the input magnitude. Using a numerical brain phantom, they show that complex-valued NNs outperform 2-channel real valued networks in the majority of their experiments, suggesting the inclusion of phase information aids in the reconstruction. Complex-valued NNs have not been trialed for cardiac MRF and the adaptation of previous methods to include complex-values could lead to better pattern matching algorithms for cardiac MRF where data is highly undersampled.

While each of the networks architectures shown here have promising features that could be employed in cardiac MRF pattern matching, most of these networks, with the exception of the work from Hamilton et al. (82), are for non-cardiac MRF sequences. Further work is needed to extend these networks to cardiac MRF where the additional dependence of measured fingerprints on the subject's cardiac rhythm during the scan must be considered. Additionally, many studies did not evaluate their networks on *in-vivo* data but instead on simulated signals (59, 93, 98), while some networks depended upon partially reconstructed data for their inputs (83, 86, 87, 89), others took data corrupted by undersampling artifacts (82, 84, 90, 95). Furthermore, most networks focused on fingerprint-wise reconstruction (59, 82–84, 86, 87, 93, 97, 98), only a few of the studies took advantage of the fact signals from neighboring tissues are correlated due to undersampling by using spatial-temporal networks (89, 90, 95). These points, combined with the fact that each study considered a different sequence, makes it hard to compare the performance of the different networks. Ideally a standardized MRF-specific dataset could be used for comparison across models.

### Sequence optimization

AI has already been used in different stages of sequence optimization in MRI, such as automatic generation of sequences (99, 100), or in the search of more efficient sampling patterns (101–104). In the field of MRF, due to the inherent flexibility of its sequence design, there are essentially infinite combinations of parameters such as flip angle trains, TR, TE, number of RF shots, position and duration of magnetization preparation pulses, and strength and waveform of gradients. As a result, most of the cardiac MRF sequences proposed so far have been designed heuristically.

An MRF sequence can be optimized in search of different goals, such as better encoding power, higher accuracy, or shorter scan times (i.e., higher efficiency). To quantify the performance of a certain sequence on these areas, different specific cost functions may be employed. The goal of a sequence optimization strategy would then be the minimization of the chosen cost function.

Given that parametric mapping in MRF is widely achieved by pattern matching between the undersampled fingerprint and the predicted dictionary, most optimization strategies have focused on the minimization of the inner product between these two signals (i.e., maximization of the encoding capability of the sequence). Cohen et al. (105) explore four different optimization algorithms to optimize the pattern of short TR and FA trains in a constrained range. Sommer et al. (106) also investigate the encoding capability of MRF sequences by inner product minimization and a Monte Carlo simulation that tries to consider the aliasing noise present in pattern matching by adding Gaussian noise to the fingerprints. Noise and aliasing artifacts were also taken into consideration in the optimization approach proposed by Kara et al. (107).

MRF sequence performance can be analyzed in terms of a cost function based on CRLB. This statistical tool looks for the lower bound of the variance of unbiased estimators and has been already utilized by MRF community to optimize FA and TR patterns for optimal sequence design (108–111). Apart from statistical-based optimizers, physics knowledge could be also included in the model, as in Jordan et al. (112).

All these algorithms work on the premise of a cost function or optimizer that is iteratively minimized. However, this is a computationally expensive task, and AI offers the ability of speeding up this process. The RNN proposed by Liu et at. (63) for dictionary generation is also employed to develop a computationally efficient method to solve the CRLB optimization. In their work, they optimize a flip angle train of an MRF sequence given two target tissues by computing the 14,000 necessary magnetization signals and their derivatives with their proposed RNN in ~10s, a reduction of two orders of magnitude in runtime. NNs and supervised learning have also been proposed for use in the joint sequence optimization and image reconstruction frame for MRI [Loktyushin et al. (113)]. However, most of these works have been proposed on phantom and *in vivo* brain MRF studies. Although efforts have been made to optimize a 2D cardiac MRF acquisition pattern (21) from a large number of simulated sequences, AI is yet to be fully exploited for sequence optimization in cardiac MRF. Particular problems within cardiac MRF sequence optimization, like RR interval dependance or short acquisition windows, are potential issues to be addressed with NN-based sequence optimization algorithms and further investigation is required for this purpose.

## Discussion

### Current limitations

The possibilities and promising results shown in recent years demonstrate that current advances in AI could become part of the cardiac MRF workflow in the near future and help its potential clinical adoption. However, there are still several remaining challenges and limitations that need to be addressed and understood before widespread implementation.

#### Data availability

The main obstacle that DL-derived techniques face in medical imaging in general is data availability. The accuracy of DL alg orithms heavily relies on the amount of data used to train and validate these algorithms. Whereas, very large databases (in some cases, containing millions of samples) can be generated in other fields in AI, the amount of trainable data available in CMR is several orders of magnitude smaller. Huge efforts are underway to generate large databases of CMR datasets, for example UK biobank or other open-source datasets (114). However, libraries of multiparametric co-registered scans (including k-space data) such as those that could be obtained with MRF are yet to be constructed, especially since multiparametric quantitative MR is not routinely carried out in a clinical environment and k-space data is not conventionally stored. Still, for AI applications to be of help in the clinical routine, training databases should include not only healthy subject data, but also incorporate multiparametric maps corresponding to different pathologies. Currently, most cardiac MRF studies in the literature are evaluated against healthy subjects or a reduced cohort of patients, although Hamilton et al. (115) recently presented results clinically evaluating a specific T_1_ and T_2_ cardiac MRF sequence. Nonetheless, this study was carried out on a relatively small number of subjects (*n* = 68) within a reduced age range. Cardiac MRF is further complicated by its dependence on subject-specific cardiac rhythms during the scan. This introduces an additional requirement for training data to either sample a wide range of heart rates and variabilities or the use of methods that rely on additional simulated data across this parameter space. Crucially, further studies are needed to generate larger clinical datasets.

The training dataset must not only be generalizable against different diagnoses but must also be unbiased. Recent studies have shown that the existence of imbalanced data may lead to inaccuracies and underperformance over different population groups, such as gender or race (116). Future studies such as that of Puyol-Anton et al. (117) should be conducted to further investigate the impact of biases and potential strategies to address this so-called “fairness”, or lack thereof, in DL.

In addition to data availability and the issue of balanced multicenter multiparametric training data, anonymization and data protection are required conditions for the use of medical data in research. These necessary privacy requirements hinder sharing of locally generated data between different medical institutions, leading to data silos. Model-sharing alternatives like the distributed DL techniques proposed by Chang et al. (118) and federated learning (118, 119), where algorithms are trained without exchanging training data from different centers, need to be explored further.

#### Reconstruction quality and fidelity

Given the lack of data availability, it is currently inevitable that many DL-MRF reconstruction approaches are based on simulated datasets. Nevertheless, for some applications, such as DL for dictionary generation (where data can be generated by EPG calculations or Bloch equations) this may be adequate. In such cases, close attention needs to be paid to the accuracy of the generated data, and its similarity to real data. The simulated data should ideally include all sort of possible imperfections that could be present in a real MRF acquisition. This implies approximations such as perfect slice profile, field homogeneity or hard RF excitation pulse should be disregarded, and instead all the possible corrections should be included, even at the cost of longer simulation times. Moreover, cardiac MRF-specific features such as cardiac and respiratory motion, mis-triggering, and an essentially infinite number of heart rate possibilities must be taken into account in the simulations. In any case, as in any other AI-based solution proposed for CMR, the algorithms need to be generalizable in different clinical settings such as vendors or field strengths, and for this to happen wide multi-vendor involvement is required.

#### Interpretability

Although novel DL algorithms have been shown to outperform non-AI-based techniques, and results may be more accurate in terms of quantitative metrics, it is often difficult to understand how these predictions have been made and where they come from. There are efforts to improve interpretability, such as *explainable AI* (120), which tries to generate solutions that can be more easily understood by the end user. However, this so-called black-box problem (121) still needs to be addressed so that the community can better comprehend what factors contribute to the decision-making of a ML model in order to better interpret its outputs.

### Future perspective

In addition to the MRF-specific solutions that the presented NNs offer, the advances in other more “traditional” directions need to also be integrated in the cardiac MRF framework. In this way, DL-based approaches for motion correction or fast CMR acquisition and reconstruction, along with the inherent versatility of cardiac MRF, would enable the extension of cardiac MRF in the spatial (from 2D to 3D), temporal (from ECG-triggered to motion-corrected free-running) and contrast (other parametric maps apart from T_1_ and T_2_) dimensions.

In addition to the usual symbiosis between cardiac MRF and ML, where ML is used as a tool to address problems faced in cardiac MRF, such as sequence optimization and reconstruction, this synergy can also be flipped and cardiac MRF could be used as a tool in a ML-based field. There has been growing interest in the use of multiparametric MRI to generate ML-based risk prediction or stratification. Radiomics is an example in this field (122), an approach where a set of medical images are used as input to extract quantitative information by means of a set of well-defined mathematical operations that are performed to extract information of the distribution and neighborhood relations of each pixel in the image of interest. These relations, or features, can be used to feed an ML algorithm and provide a quantitative analysis. Radiomics is a well stablished technique in some medical imaging fields like oncology, and it has started showing its potential for MRI and particularly CMR application in the recent years (123), however reproducibility is still limited.

Conventionally, the input datasets used for CMR radiomics are Late-Gadolinium Enhanced (LGE), CINE or contrast-weighted semi-quantitative images (124–128). However, in recent years quantitative information given by parametric maps such as T_1_ and T_2_ have been employed, showing a great potential (129–133). In most of these studies, only one type of relaxation parameter is used for radiomics analysis. Nevertheless, as in conventional quantitative CMR, stacking multiparametric information could increase the diagnostic power of radiomics, as shown by Baessler et al. (129, 130). However, an accurate and robust multiparametric radiomics analysis can only be performed when the different parametric maps are perfectly aligned. This is where the multiparametric information provided by cardiac MRF could become a more robust input for radiomics applications, due to its inherent spatial and temporal co-registration. Consequently, cardiac MRF presents the potential to generate a multidimensional dataset that may serve as an input to improve diagnostic capacity of radiomics or other DL approaches for diagnosis in CMR.

## Conclusion

Cardiac magnetic resonance fingerprinting is increasingly proven in its potential as a valuable tool for multiparametric quantification on CMR. Its scalable ability to generate several parametric maps within the same acquisition usually comes, however, at the cost of sequence complexity and increased reconstruction and dictionary generation times. This is further aggravated by the CMR-specific problems, such as unpredictable heart rate and cardiac motion. Nevertheless, recent advances in AI applied to medical imaging have shown that, with the correct understanding of the type of network required for the specific problem and a sufficient amount of training data, NNs are capable of solving many of these problems, much more rapidly and to comparable accuracy as conventional methods. Thus, the field of AI, which has experienced a rapid growth in recent years, is expected to become part of cardiac MRF at every step of its framework (sequence optimization, dictionary generation, image reconstruction, parametric estimation and analysis) and greatly contribute to the potential inclusion of cardiac MRF in the clinical routine. Nonetheless, special care needs to be taken to overcome the limitations that may hinder this goal; aspects such as algorithm interpretability and most importantly data availability need to be enforced to ensure AI is used at its full capacity in cardiac magnetic resonance fingerprinting.

## Author contributions

CV and TF devised and wrote the manuscript. RB and CP reviewed the manuscript. All authors contributed to the article and approved the submitted version.

## Funding

The authors acknowledge financial support from the BHF PG/18/59/33955 and RG/20/1/34802, EPSRC EP/V044087/1, EP/P001009, EP/P032311/1, EP/P007619, Wellcome EPSRC Center for Medical Engineering (NS/A000049/1), Millennium Institute for Intelligent Healthcare Engineering ICN2021_004, FONDECYT 1210637 and 1210638, ANID - Basal FB210024, Millenium Nucleus NCN19_161, and the Department of health *via* the National Institute for Health Research (NIHR) comprehensive Biomedical Research Center award to Guy's and St. Thomas' NHS Foundation Trust.

## Conflict of interest

The authors declare that the research was conducted in the absence of any commercial or financial relationships that could be construed as a potential conflict of interest.

## Publisher's note

All claims expressed in this article are solely those of the authors and do not necessarily represent those of their affiliated organizations, or those of the publisher, the editors and the reviewers. Any product that may be evaluated in this article, or claim that may be made by its manufacturer, is not guaranteed or endorsed by the publisher.

## Author disclaimer

The views expressed are those of the authors and not necessarily those of the NHS, the NIHR, or the Department of Health.
